# A Progressive Loss Decomposition Method for Low-Frequency Shielding of Soft Magnetic Materials

**DOI:** 10.3390/ma17225584

**Published:** 2024-11-15

**Authors:** Airu Ji, Jinji Sun

**Affiliations:** 1 Key Laboratory of Ultra-Weak Magnetic Field Measurement Technology, Ministry of Education, School of Instrumentation and Optoelectronic Engineering, Beihang University, Beijing 100191, China; jiairu@buaa.edu.cn; 2 Zhejiang Provincial Key Laboratory of Ultra-Weak Magnetic-Field Space and Applied Technology, Hangzhou Innovation Institute, Beihang University, Hangzhou 310052, China

**Keywords:** statistical theory of losses, low-frequency magnetic shielding, soft magnetic materials, progressive loss decomposition

## Abstract

Energy loss in shielding soft magnetic materials at low frequencies (1–100 Hz) can cause fluctuations in the material’s magnetic field, and the resulting magnetic noise can interfere with the measurement accuracy and basic precision physics of biomagnetic signals. This places higher demands on the credibility and accuracy of loss separation predictions. The current statistical loss theory (STL) method tends to ignore the high impact of the excitation dependence of quasi-static loss in the low-frequency band on the prediction accuracy. STL simultaneously fits and predicts multiple unknown quantities, causing its results to occasionally fall into the value boundary, and the credibility is low in the low-frequency band and with less data. This paper proposes a progressive loss decomposition (PLD) method. Through multi-step progressive predictions, the hysteresis loss simulation coefficients are first determined. The experimental data of the test ring verifies the credibility of PLD’s prediction of the two hysteresis coefficients, improving the inapplicability of the STL method. In addition, we use the proposed method to obtain the prediction results of the low-frequency characteristics of the loss of a variety of typical soft magnetic materials, providing a reference for analyzing the loss characteristics of materials.

## 1. Introduction

Fields such as biomagnetic signals, basic precision physics and aerospace require the use of magnetic shielding devices to shield low-frequency (1–100 Hz) geomagnetic interference. Current low-frequency geomagnetic shielding devices are often made of soft magnetic materials with high magnetic permeability. Internal mechanisms such as the magnetic domain deflection of high-permeability soft magnetic materials will cause fluctuations in the material’s magnetic field. According to the fluctuation dissipation theorem, which uses the fluctuation of the system in thermal equilibrium to represent the linear response to external disturbances, we can characterize the level of magnetic disturbance by the energy loss level [1]. This will produce a background noise of the precision test instruments, such as atomic gyroscopes [2] and atomic magnetometers [3,4]. The fluctuations caused by the loss characteristics of shielding materials also restrict the accuracy of biomagnetic measurements, such as magnetoencephalography (MEG) and magnetocardiography (MCG) [5,6]. Murger et al. proposed the use of low-loss ferrite materials to reduce the magnetic fluctuations of shielding [7]. Many studies have proven the rationality of using low-loss soft magnetic materials for geomagnetic low-frequency shielding [8,9]. This puts forward new requirements for the analysis of low-frequency loss characteristics of various soft magnetic materials. Using loss analysis and separation methods, the proportion of various loss mechanisms of materials can be analyzed.

Currently, soft magnetic materials with low coercivity are widely used in power electronics and motors, where their main function is to distribute electrical energy. These fields place precise test requirements on the loss performance of materials in the high frequency band (kHz–MHz). For example, ferrites are often used in transformer cores, input filters, resonant circuit inductors and output chokes [10,11,12]. These fields require MnZn ferrites or Ni-Zn ferrites to show low energy losses in the 1–1000 kHz and 1–1000 MHz frequency bands and at high induction levels [13], respectively. In addition to well-known materials such as ferrites and silicon steel, there is also a relatively niche but important market for soft magnetic specialty alloys [14,15]. Both amorphous cobalt-based alloys and nanocrystalline iron-based alloys have high permeability and low coercivity [16], and exhibit low losses in the kilohertz and megahertz frequency ranges. They have been shown to be competitive with conventional silicon steel and ferrites in high-frequency bands [17].

At present, the study of the loss characteristics of soft magnetic materials often ignores the influence of the excitation dependence of quasi-static loss on the calculated hysteresis loss. This is because the study of soft magnetic materials is usually carried out in the higher frequency range, and such influence can be ignored. Lauda et al. studied the soft magnetic material FeSi/MnZnFe_2_O_4_ in the frequency range of 1–13 kHz [18]. Ibrahim et al. studied the loss characteristics of soft magnetic materials in motors in the 4000 Hz frequency band and proposed a loss separation method [19]. Kollár et al. studied two Fe-based soft magnetic composite materials at 1 kHz, using Bertotti’s statistical theory [20].

The field of low-frequency (1–100 Hz) geomagnetic shielding has put forward the requirement for the accurate testing of the low-frequency loss characteristics of shielding materials. Many authors have used the conventional three-stage single-step prediction method in the field of low-frequency shielding [5,21]. However, in the low-frequency band, the excitation dependence of quasi-static loss will affect the prediction accuracy. In addition, the conventional STL method is prone to overfitting or falling into local optimum for loss data with a small amount of data, which will lead to inaccurate material property analysis.

In this paper, we propose a progressive loss decomposition (PLD) method. In Section 2, we present the theoretical analysis of the PLD method. In Section 3, we present the test instrument and the PLD method, and compare the results of PLD and the conventional STL method. In Section 4, we perform the low-frequency loss prediction and analysis of four typical soft magnetic materials using PLD.

## 2. Theory and Analysis

There are three classes of models for material loss separation, each offering a different tradeoff between accuracy and computational complexity: The first is the Steinmetz equation [22]. Its advantages are simplicity and speed. But it does not apply to some magnets. The second is Dynamic Preisach model (DPM) [23]. It takes the skin effect into account, but requires a lot of calculations [24,25]. It is not applicable to the research object of this paper. The third is Loss Statistical Theory (STL) [26]. The loss per cycle *W* at any frequency under any induction waveform can be decomposed as $W=W_{h}+W_{e}+W_{\mathrm{exc}}$, where $W_{h}$ is the hysteresis loss, $W_{e}$ is the eddy current loss, and $W_{\mathrm{exc}}$ is the residual loss. The formula can be modified to adapt to different situations, such as inductive distortion [27,28].

We propose an improved loss separation method to improve the calculation accuracy in the low-frequency band, combining the Steinmetz equation and STL. The STL formula includes classical losses, arising from macroscopic large-scale behavior, and hysteresis losses, originating from microscopic discontinuous magnetization processes, which are proportional to ${\left(B_{m}\cdot f\right)}^{2}$ and $B_{m}^{2}\cdot f$ expressed in power, where $B_{m}$ is the peak magnetic induction [29]. Whenever the characteristic time scales of these two processes do not overlap, separation can be achieved.

The intercept of the loss curve for frequency should come entirely from the quasi-static losses. Based on the Steinmetz equation for sinusoidal excitation, $P_{v}=k\cdot f^{a}\cdot B_{m}^{b}$, we add the quasi-static loss term $P_{\mathrm{qs}}$ to the frequency-dependent prediction. Quasi-static losses are the frequency-independent part of the hysteresis losses: $P_{\mathrm{qs}}=C_{h}\cdot B_{m}^{\alpha }$ with a $B_{m}$ dependence, where the power α is a coefficient in the STL formula that depends on the material properties. Therefore, α can be fitted by

(1) $$\left(\frac{P_{v}}{f}\right){|}_{f=0}=C_{h}\cdot B_{m}^{\alpha }.$$

Then, we use the STL prediction method to predict the classic energy loss [30]. In addition to hysteresis loss $P_{h}$, dynamic loss also includes classical loss, most of which comes from eddy current loss $P_{e}$. The part of dynamic loss caused by the domain effect that is greater than $P_{e}$ is called the excess loss $P_{\mathrm{exc}}$, which is greater than $P_{e}$ in many cases.

In the case where skin effect can be neglected, eddy current loss can be represented as

(2) $$P_{e}=\frac{\sigma d^{2}f}{12}\int_{0}^{f^{-1}}{\left[\frac{d(B_{m}\sin\left(2\pi ft\right))}{dt}\right]}^{2}dt=C_{e}B_{m}^{2}f^{2},$$

where σ is the material conductivity, *d* is the thickness of the sheet, *f* is the magnetization frequency, B(t) is the instantaneous magnetic flux density, and $C_{e}=\frac{\sigma d^{2}\pi^{2}}{6}$ is the eddy current loss coefficient, obtained by fitting when the parameters are unknown.

Residual loss can be expressed as

(3) $$P_{\mathrm{exc}}=\sqrt{\sigma GSV_{0}}\cdot f\cdot \int_{0}^{f^{-1}}{\left|\frac{dJ}{dt}\right|}^{1.5}dt=C_{\mathrm{exc}}B_{m}^{1.5}f^{1.5},$$

where the polarization intensity $J=B-\mu_{0}H$, *S* is the cross-sectional area of the stack, G=0.1356 is a dimensionless coefficient, and $V_{0}$ is a statistical parameter related to the distribution of local coercive fields. Some authors have applied the Static Preisach Model (SPM) to calculate $P_{h}$, but their simplified model neglects the dependence of $P_{\mathrm{exc}}$ on a DC bias. This approximation leads to a small error, but it is physically inadequate [31].

If further separation and analysis of eddy current loss are required, we can consider inter-particle loss $P_{\mathrm{inter}}$ (loss caused by eddy current flowing along a cross-section perpendicular to the magnetic flux) and intra-particle loss $P_{\mathrm{intra}}$ (loss caused by eddy current flowing inside the particle—due to the small average particle size, we ignore the skin effect) [32]. For the case of absolute insulation between particles, $P_{\mathrm{inter}}=0$ and $P_{\mathrm{intra}}=1$. Ignoring the influence of particles, $P_{\mathrm{inter}}=1$ and $P_{\mathrm{intra}}=0$. Since this paper does not study the loss of powder materials, it will not be described in detail here.

## 3. Experiment and Method Comparison

We used the experimental data of Permalloy 1j85 to illustrate the detailed steps. The dimensions of the Permalloy rings are shown in Section 4. First, we needed to obtain the dynamic loss data of the material of the magnetic ring through testing methods.

The international standard IEC60404-6 [33] specifies the test method for AC magnetic characteristics. The AC magnetization curve and hysteresis loop were also based on the principle of electromagnetic induction, and the dynamic magnetic characteristics were measured by the volt–ampere method, i.e., by applying AC excitation magnetic fields of different amplitudes and frequencies, measuring the induced electromotive force, and then obtaining the AC magnetic parameters.

**Figure 1:**
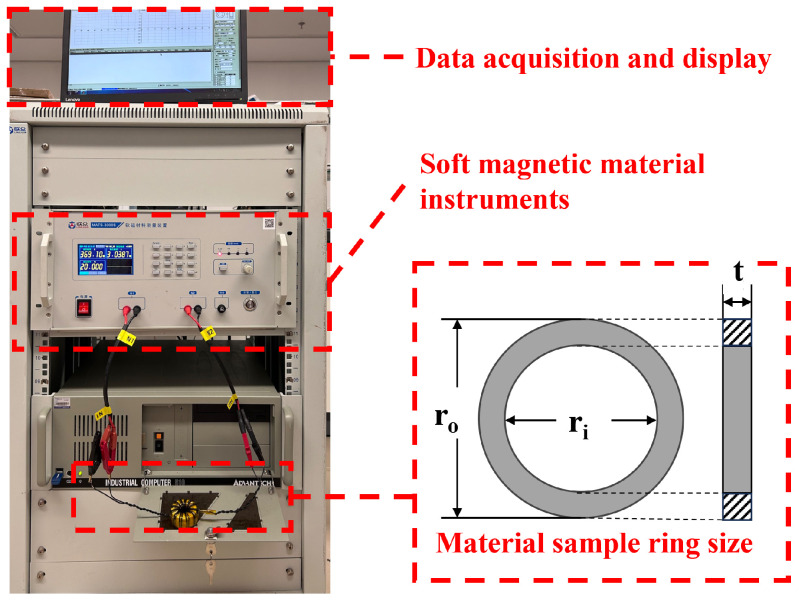
Test instrument and magnetic ring size.

We used the material’s magnetic property measuring instrument (Hunan linkjoin Technology, Loudi, China, MATS-3010SA) to measure the AC magnetic properties of the sample ring, as shown in Figure 1. We conducted the test at room temperature of 23–25 degrees Celsius in a geomagnetic environment. Slight fluctuations in room temperature within this range would not have a significant impact on the test results [34]. We measure the outer diameter $r_{o}$, inner diameter $r_{i}$, thickness *t* and mass *m* of the sample ring. The above physical quantities are all in international standard units. Given the excitation frequency and maximum magnetic flux $B_{m}$, we obtained the total loss $P_{s}$ of the magnetic ring through instrument testing. The accuracy standard of the MATS-3010SA is as follows: For the frequency range of 10–20 kHz, the uncertainty of measuring $P_{s}$ of the permalloy ring sample is 3%; the uncertainty of measuring $P_{s}$ of the amorphous ring sample is 3%; and for the ferrite ring sample, it is 5%. The unit volume loss of the magnetic ring is

(4) $$P_{v}=\frac{4P_{s}}{\pi t\left(r_{o}^{2}-r_{i}^{2}\right)}.$$

**Figure 2:**
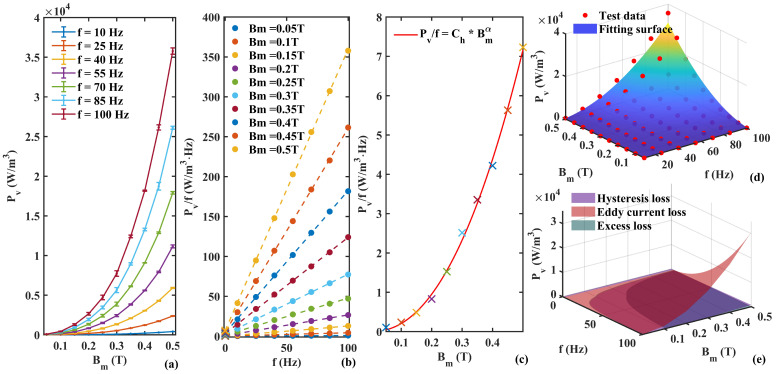
Loss separation process. (**a**) Original test data (including error bars). (**b**) First step fitting. (**c**) Second step fitting: intercept of each curve in (**b**). The color of symbol ’x’ corresponds to the legend in (**b**). (**d**) Total loss fitting surface: the fitting surface uses parula colormap to represent the size of the predicted value. (**e**) Loss separation prediction results.

Figure 2 shows the steps of the improved loss separation method PLD. Figure 2a shows the 1–100 Hz loss test results of Permalloy 1j85 from multiple measurements. The coefficients that need to be determined are $C_{h}$, α, $C_{e}$, $C_{\mathrm{exc}}$. The total loss obtained by PLD is $P_{\mathrm{vp}}=C_{h}B_{m}^{\alpha }f+C_{e}B_{m}^{2}f^{2}+C_{\mathrm{exc}}B_{m}^{1.5}f^{1.5}$.

The first step is shown in Figure 2b, where we used a multinomial power function of frequency to predict the ratio of the total power loss per unit volume of the material to the frequency $P_{v}/f$ under different $B_{m}$ values. Since classical losses only occur under alternating conditions, the intercept of its loss value that extrapolated to zero frequency should be entirely composed of hysteresis losses, as shown in Formula (1). The zero frequency intercept is represented by a cross.

In the second step shown in Figure 2c, the intercept obtained in the previous step is fitted using $C_{h}B_{m}^{\alpha }$ to obtain the values of the hysteresis loss coefficient $C_{h}$ and the frequency-dependent power α. The first determination of two parameters avoids the fitting situation of four unknowns.

The third step is to use the total loss data to predict the remaining unknown parameters $C_{e}$ and $C_{\mathrm{exc}}$. Then, the total predicted loss $P_{\mathrm{vp}}$ can be obtained in Figure 2d. The surface formula of total loss with frequency and $B_{m}$ is obtained, and the prediction accuracy with the test data points is characterized by the prediction error:

(5) $$e_{p}=\frac{SS_{\mathrm{res}}}{SS_{\mathrm{tot}}}=\frac{\Sigma {\left(P_{v}-P_{\mathrm{vp}}\right)}^{2}}{\Sigma {\left(P_{v}-\bar{P_{v}}\right)}^{2}}.$$

In the fourth step, we plotted the separation of $P_{\mathrm{vp}}$ generated by different mechanisms in the material magnetic ring experiment, including eddy current loss, hysteresis loss, and excess loss. This important conclusion reflects the loss of various mechanisms of the material.

This loss decomposition method consists of multiple progressive prediction steps, called the progressive loss decomposition (PLD) method. Table 1 compares the prediction results of the PLD method in this paper and the conventional STL three-stage single-step prediction method for the Permalloy data. The STL method firstly requires the material-dependent Steinmetz constant α of Permalloy, which is determined as 2.5.

The test results show that PLD and STL have similar fitting errors for total loss fitting, which means that both methods have certain effects on total loss fitting. However, STL needs to fit four unknown parameters at the same time, which is too dependent on the accuracy of the model. In practical applications, our researchers found that the results of STL are occasionally not reliable enough. For the test data of some materials, the prediction results of the alpha parameter by the STL method are often highly correlated with the given value range. For example, for this group of Permalloy test data, for a given alpha coefficient, different value ranges will lead to large differences in the prediction results. The alpha fitting result falls on the value boundary. We can only query the α=2.5 coefficient of Permalloy first, and then compare it with the fitting results.

Therefore, when this phenomenon occurs when using STL, it is more effective to use the PLD method proposed by us instead. The prediction accuracy of PLD for alpha and Ch is more trustworthy and less dependent on the accuracy of the model.

## 4. Multi-Material PLD Results

We use the PLD method to separate and predict the losses of four low-frequency shielding soft magnetic materials, including (a) 1j85 Permalloy of the General Iron and Steel Research Institute, (b) Co-based amorphous alloy 2714 (${\mathrm{Co}}_{66}{\mathrm{Fe}}_{4}{\mathrm{Ni}}_{1}B_{14}{\mathrm{Si}}_{15}$), (c) Finemet-type nanocrystalline alloy, and (d) MnZn Ferrite DMR95. Table 2 shows the parameters of magnetic rings made of four materials.

**Figure 3:**
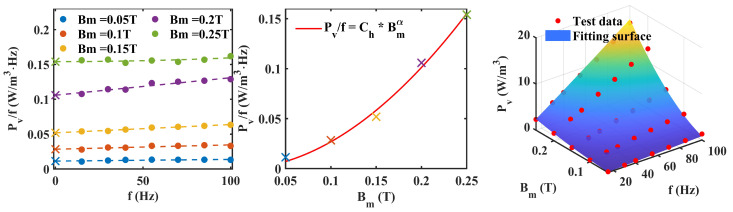
Loss separation prediction steps for Co-based amorphous. (The symbol ‘x’ represents the intercept of each curve. The fitting surface represents the size of the predicted value using parula colormap).

**Figure 4:**
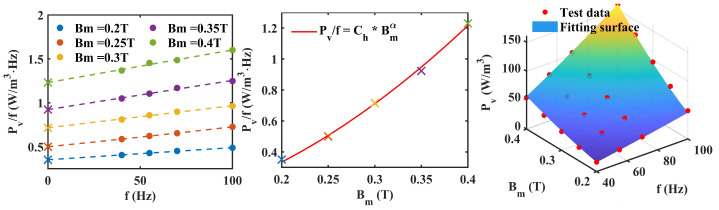
Loss separation prediction steps for Finemet NANO. (The symbol ‘x’ represents the intercept of each curve. The fitting surface represents the size of the predicted value using parula colormap).

**Figure 5:**
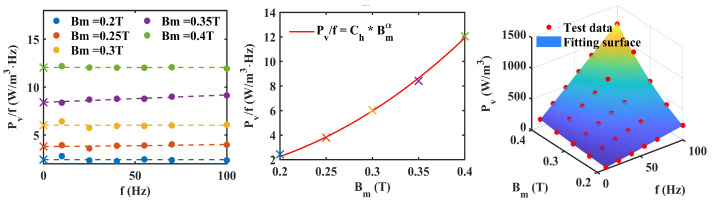
Loss separation prediction steps for ferrtie. (The symbol ‘x’ represents the intercept of each curve. The fitting surface represents the size of the predicted value using parula colormap).

The improved method proposed in Section 2 is used for Co-based amorphous, Finemet-NANO and ferrite materials to separate different losses. Figure 3, Figure 4 and Figure 5 show the loss analysis process of the three materials. It is worth noting that the α of amorphous and nanocrystalline is lower than that of other materials, which means that the frequency dependence of their hysteresis loss is more gentle. Their coefficients are also relatively low, resulting in low loss characteristics. The $C_{e}$ of ferrite is lower than that of the other three materials, which means that it has low eddy current loss characteristics.

Table 3 shows the results of PLD and STL for the same test data of several materials. Among them, the STL method fits and predicts four unknown quantities at the same time, the fitting range of α is fixed to 1–3, and the fitting range of other parameters is 0-inf. We retain the precision to four significant digits.

**Figure 6:**
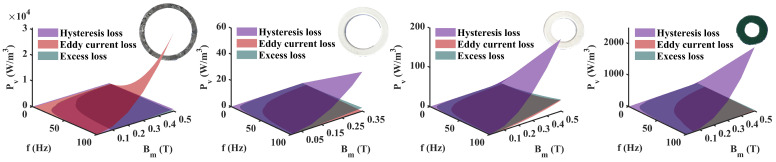
Separation of power loss components of 4 materials.

Using the coefficients to perform a three-dimensional loss separation on the four materials, we can obtain the loss results of different mechanisms in fixed frequency bands and magnetic field bands. The fourth step in Figure 2 shows the results for Permalloy 1j85, and Figure 6 shows the results for the remaining three materials.

Four representative geomagnetic shielding materials each have significant differences in loss mechanisms. Based on the frequency-dependent three-dimensional loss separation of different mechanism losses, the results show significantly different loss characteristics. This is mainly reflected in the different dominance of eddy current loss and hysteresis loss, which are closely related to material properties.

The eddy current loss of Permalloy 1j85 is higher than the hysteresis loss, while the hysteresis loss of Co-based amorphous, Fe-based nanocrystals and ferrites is higher than the eddy current loss. Co-based amorphous and Fe-based nanocrystals have similar performances, with higher permeability and lower total loss than Permalloy and ferrite. The high resistivity of ferrites results in the smallest eddy current loss share. The loss separation results reflect the performance of different materials under different loss mechanisms.

## 5. Conclusions

Loss separation is of great significance for distinguishing the distribution of noise sources in soft magnetic materials. According to relevant research at Princeton University [35], the material loss mechanism is closely related to the material magnetic noise mechanism, so the total power loss can qualitatively represent the relative magnetic noise of different materials, and the loss prediction results can provide data support for subsequent magnetic noise analysis. In addition, the test results guide the specific selection and processing of materials in subsequent applications. The low saturation magnetization of ferrites hinders their effective operation under high external magnetic fields. In contrast, Permalloy exhibits high saturation magnetization but high eddy current loss, resulting in high total loss at higher operating frequencies. Although cobalt-based amorphous and iron-based nanocrystals have better loss performance, the low strength of the material makes stress and damage fatal to their performance. For Permalloy, slicing or adding an insulating layer can be used to increase the resistivity, but note that this will also cause some performance degradation, such as permeability or saturation magnetization.

It should be noted that this paper focuses on the loss analysis of the material state when used for low-frequency shielding, aiming to provide reference for researchers related to shielding. For Permalloy, when used for shielding, it is often processed into 1–5 mm sheets or spliced sheets. Co-based amorphous and nanocrystalline are often processed into multi-layer strip bonding for shielding. Since eddy current loss is highly related to the material state (strip, block or powder) and thickness, the eddy current loss performance of materials of different thicknesses cannot be directly compared. Therefore, this article only provides a quantitative comparison of the losses of magnetic rings made of different materials, rather than a performance comparison of the materials themselves.

## Figures and Tables

**Table 1:** Parameters of power loss separation process.

Method	PLD	STL
α	2.204	2.500
$C_{h}$	33.03	362.10
$R^{2}\left(P_{v}/f\right)$	99.82%	-
$C_{e}$	12.24	9.780
$C_{\mathrm{exc}}$	8.584 × 10^−7^	6.818 × 10^−14^
$e_{p}$	2.94%	3.08%

**Table 2:** Material sample ring size specification.

Material	Permalloy	Co-Based Amorphous	Finemet-NANO	Ferrite
Outside diameter $r_{o}$ (mm)	40	36	25	20
Inner diameter $r_{i}$ (mm)	32	25	20	10
Thickness *t* (mm)	3	11	5	7.3
Quality *m* (g)	10.88	15.87	2.81	8.55
Averaged crystallite size (nm)	-	8	9	-
Lamination thickness	1 mm	20 μm	20 μm	-

**Table 3:** Comparison of magnetic ring loss separation results of three materials.

Method and Materials	PLD	STL	PLD	STL	PLD	STL
	Co-Based Amorphous		Finemet-NANO		Ferrite	
α	1.915	1.697	1.872	1.890	2.408	2.383
$C_{h}$	2.204	1.449	6.758	5.313	108.3	56.43
$R^{2}\left(P_{v}/f\right)$	99.40%	-	99.75%	-	99.80%	-
$C_{e}$	4.482 × 10^−11^	4.583 × 10^−7^	0.01028	5.017 × 10^−12^	8.274 × 10^−12^	1.358 × 10^−5^
$C_{\mathrm{exc}}$	0.01221	0.02271	0.08814	0.2580	0.09438	0.1385
$R^{2}\left(P_{v}\right)$	98.89%	98.67%	99.73%	99.95%	99.91%	99.90%

## Data Availability

The original contributions presented in the study are included in the article, further inquiries can be directed to the corresponding author.
